# Analysis of the Association Between *TERC* and *TERT* Genetic Variation and Leukocyte Telomere Length and Human Lifespan—A Follow-Up Study

**DOI:** 10.3390/genes10020082

**Published:** 2019-01-25

**Authors:** Daniela Scarabino, Martina Peconi, Franca Pelliccia, Rosa Maria Corbo

**Affiliations:** 1 CNR Institute of Molecular Biology and Pathology, P.le Aldo Moro 5, 00185 Rome, Italy; daniela.scarabino@cnr.it; 2 CNR Institute of Translational Pharmacology, Via Fosso del Cavaliere 100, 00133 Rome, Italy; martinapeconi@gmail.com; 3 Department of Biology and Biotechnology, La Sapienza University, P.le Aldo Moro 5, 00185 Rome, Italy; franca.pelliccia@uniroma1.it

**Keywords:** human lifespan, genetic variation, *TERC*, *TERT*, leukocyte telomere length

## Abstract

We investigated the possible influence of *TERC* and *TERT* genetic variation and leukocyte telomere length (LTL) on human lifespan. Four polymorphisms of *TERT* and three polymorphisms of *TERC* were examined in a sample of elderly subjects (70–100 years). After nine years of follow-up, mortality data were collected, and sub-samples of long-lived/not long-lived were defined. *TERT* VNTR MNS16A L/L genotype and *TERT* rs2853691 A/G or G/G genotypes were found to be associated with a significantly higher risk to die before the age of 90 years, and with a significantly lower age at death. The association between lifespan and LTL at baseline was analyzed in a subsample of 163 subjects. Age at baseline was inversely associated with LTL (*p* < 0.0001). Mean LTL was greater in the subjects still living than in those no longer living at follow-up (0.79 T/S ± 0.09 vs. 0.63 T/S ± 0.08, *p* < 0.0001). Comparison of age classes showed that, among the 70–79-year-olds, the difference in mean LTL between those still living and those no longer living at follow-up was greater than among the 80–90-year-olds. Our data provide evidence that shorter LTL at baseline may predict a shorter lifespan, but the reliability of LTL as a lifespan biomarker seems to be limited to a specific age (70–79 years).

## 1. Introduction

The dramatic increase in rates of survival to an advanced old age over the past century has prompted extensive research in the attempt to identify the mechanisms involved in lifespan determination. Among the most extensively studied biological processes associated with longevity are those involved in cell maintenance/senescence. Telomeres, the structures at the ends of eukaryotic chromosomes with a protective action against genome instability, have been widely studied as a possible determinant of lifespan [1]. Human telomeres are composed of repeated TTAGGG nucleotide sequences located at the ends of each chromosome. Because telomere sequences are not fully replicated during DNA replication due to the inability of DNA polymerase to replicate the 3′ end of the DNA strand, telomeres shorten as cells divide. In the absence of special telomere maintenance mechanisms, telomeres (and chromosomes) become shorter with each cell division. Once a critically short telomere length is reached, the cell is triggered to enter replicative senescence, ultimately leading to cell death. Telomerase, a cellular ribonucleoprotein enzyme complex, counteracts telomere shortening [2]. Human telomerase is constituted by a DNA reverse transcriptase polymerase (telomerase reverse transcriptase, *TERT*), which uses an RNA template (telomerase RNA component, *TERC*) to add telomeric DNA onto telomeres, thus compensating for the telomere shortening caused by cell divisions [3]. The two components of human telomerase are encoded by the *TERT* gene on 5p15.33 (OMIM:187270) and by the *TERC* gene on 3q26 (OMIM:602322). Since telomerase is almost totally absent in adult tissues, including the skin, kidney, liver, blood vessels, and peripheral leukocytes, the telomeres of replicating cells shorten progressively. This mechanism is thought to underlie aging and age-associated diseases [4,5,6]. Average leukocyte telomere length (LTL) is generally used as a marker of overall telomere length, since TLs have been found to be strongly correlated across different cell types within the same individual [7,8]. Population studies that have applied analysis of LTL support the hypothesis that leucocyte telomere shortening is associated with aging and lifespan [4,5,6,9,10]; however, the associations with age-related chronic diseases (cardiovascular and metabolic disease, cancer) are not always concordant [8,9,11,12].

As fully functional telomerase is critical for telomere maintenance, genetic variations of human *TERT* and *TERC* genes may alter the stability of the telomerase complex or directly affect its enzymatic activity [13]. Studies assessing the possible effect of genetic polymorphisms of human *TERT* and *TERC* genes on LTL [13,14,15,16], and on aging and lifespan [17,18,19], have produced mixed results. While some *TERC* or *TERT* SNPs were found to be associated with longevity, the relation was not always mediated by the association with telomere length. Similar contradictory results have come from genetic association studies of *TERT* polymorphisms and common diseases [8]. 

In the present study, we investigated the possible impact on the human lifespan of four polymorphisms of the *TERT* gene (MNS16A, rs2853691, rs33954691, rs2736098) and three polymorphisms of the *TERC* gene (rs12696304, rs3772190, rs16847897). MNS16A is a minisatellite (variable number of tandem repeats, VNTR) located downstream of exon 16 of the *TERT* gene and upstream in the putative promoter region of an antisense *TERT* transcript. It shows two common alleles (VNTR-302 or L and VNTR-243 or S on the basis of the PCR fragment size) [20]. It has been studied in relation to longevity and cancer risk [18,21,22,23]. The detection of antisense *TERT* mRNA suggested its possible role in regulating human telomerase expression [20]. *TERT* rs2853691 is located in an intronic region and shows two common alleles, A and G, while rs33954691 is located in exon 14, where a C to T substitution does not result in a change of the amino acid (Histidine) at codon 1013. These two *TERT* SNPs have been reported to be associated with both LTL and lifespan [13,17]. rs2736098 is located in exon 2, where a G to A substitution does not result in a change of the amino acid alanine at codon 305; it has shown a strong association with some cancer types (see OMIM %613059). The *TERC* SNPs rs12696304, rs3772190, and rs16847897 are all located downstream of *TERC* in a noncoding region [14,17] and have been consistently associated with variation of LTL [14,16,17,24]. In addition, in the attempt to gain a better understanding of the relationships between telomere length and lifespan, in the present study, we analyzed LTL in a subsample of elderly subjects who had been genotyped for *TERT* and *TERC* polymorphisms. 

The association between LTL and *TERT* and *TERC* polymorphisms and longevity was investigated by means of a follow-up study. The study sample was originally recruited in 2000. After collecting mortality information in 2009, we defined a sample of long-lived subjects as those who died after the age of 90 years, and a sample of not-long-lived subjects composed of those who had died before reaching 90 years of age.

## 2. Materials and Methods

### 2.1. Materials

The sample was recruited in 2000 for the multidisciplinary LONCILE (Longevity of Cilento) study on the anthropological and biological characteristics of the elderly population of the Cilento area in the district of Salerno, southern Italy [25]. As previously reported [26], it consisted of 277 unrelated individuals (43.7% males) born between 1900 and 1930 (mean age, 82.9 ± 5.7 years ± standard deviation [SD]), enrolled without selection criteria, except age (>70 years) and birth place; they had no manifest pathologies and were healthy, consistent with age. Mortality data on 267 subjects were collected in 2009. In 2000, 14.5% were aged 90 years old or older. During the nine-year follow-up period, the mortality rate was 62.5% (51.5% men), and 44.9% of the subjects died after the age of 90 years, including those aged 90 at baseline. As the mean life expectancy in this geographic area in 2000 for subjects 83 years old was seven years for women and six years for men (ISTAT, http://demo.istat.it/unitav/index.html), we defined as long-lived those subjects who, at follow-up in 2009, had died at an age of more than 90 years (≥90 years). The sample of the long-lived (*n* = 75) comprised 100% of subjects aged 90 years or older in 2000 and 36.3% of those aged over 80 years in 2000. The sample of the not long-lived (*n* = 89) was made up of individuals who had died between 2000 and 2009 before reaching the age of 90 years. 

The protocol for the collection of biological material for the scientific studies was approved by the institutional committees (Local Health Unit, Salerno 3). The study was approved by the Department Board (12/06/2009 session) of the former Department of Genetics and Molecular Biology of La Sapienza University, Rome. Written, informed consent was obtained from all subjects.

### 2.2. Laboratory Methods

Genomic DNA was extracted according to the salting out procedure described by Miller et al. [27] from venous blood drawn in EDTANa2 as anticoagulant from all subjects after overnight fasting.

*TERT* VNTR MNS16A was genotyped according to the allelic-specific PCR method, as previously reported [20,23]. Genotyping revealed, in addition to the most common alleles corresponding to 243 bp band and 302 bp band, less frequent but still polymorphic alleles corresponding to 274 bp band and 333 bp band. The genotypes were then classified according to Wang et al. [20]: short allele (S) corresponds to 243 and 274 bp bands and long allele (L) to 302 and 333 bp bands. The MNS16A genotypes were L/L, L/S, and S/S. *TERT* SNPs (rs2853691 and rs33954691) were investigated by polymerase chain reaction amplification followed by restriction fragment length polymorphism (PCR-RFLP), as previously reported [23]. Genotyping of the *TERT* SNP (rs2736098) and the *TERC* SNPs (rs12696304, rs3772190, and rs16847897) was carried out by allelic discrimination using predesigned TaqMan SNP genotyping assays (Applied Biosystems), as previously reported [23]. The genotyping techniques are reported in detail in Supplementary Materials (Figures S1–S3).

The average (of triplicate) telomere length in leukocytes was measured by real-time PCR quantitative analysis (qPCR) on a 7300 real-time PCR instrument (Applied Biosystems). This method allows the determination of the number of copies of telomeric repeats (T) compared to a single copy gene (S) used as a quantitative control (T/S ratio) [28]. The telomere and single-copy gene β-globin (HGB) were analyzed on the same plate in order to reduce inter-assay variability. DNA (35 ng) was amplified in a total volume of 20 μl containing 10 μl of SYBER Select Master Mix (Applied Biosystems); primers for telomeres and the single-copy gene were added to final concentrations of 0.1 μM (Tel Fw), 0.9 μM (Tel Rev), 0.3 μM (HGB Fw), and 0.7 μM (HGB Rev), respectively. The primer sequences were: Tel Fw 5′-CGGTTTGTTTGGGTTTGGGTTTGGGTTTGGGTTTGGGTT-3′; Tel Rev 5′-GGCTTGCCTTACCCTTACCCTTACCCTTACCCTTACCCT-3′; HGB Fw 5′-GCTTCTGACACAACTGTGTTCACTAGCAAC-3′; and HGB Rev 5′-CACCACCAACTTCATCCACGTTCACCTTGC-3′ [29]. The enzyme was activated at 95 °C for 10 min, followed by 40 cycles at 95 °C for 15 s and 60 °C for 1 min. In addition, two standard curves (one for HGB and one for telomere reactions), were prepared for each plate using a reference DNA sample (Control Genomic Human DNA, Applied Biosystems) diluted in series (dilution factor = 2) in order to produce five concentrations of DNA ranging from 50 to 6.25 ng in 20 µL. Measurements were performed in triplicate and are reported as the T/S ratio relative to the calibrator sample to allow for comparison across runs.

### 2.3. Statistical Analysis

Allelic frequencies were determined by the gene-counting method. Agreement between the observed genotype distributions and those expected according to the Hardy-Weinberg equilibrium was verified with a chi square test. Linkage disequilibrium (LD) between the *TERT* and *TERC* SNPs and haplotype frequencies were estimated by the maximum likelihood method using the EH program (http://www.genemapping.cn/eh.htm) [30]. The differences in allele, genotype, and haplotype frequencies between patients and controls were analyzed with a chi square test. The probability of living to an age over 90 years (≥90 years) or not associated with *TERT* genotypes was estimated by odds ratios (ORs) adjusted for other variables calculated by logistic regression. 

Parametric (ANOVA) and non-parametric (Kruskal-Wallis) tests were used to compare the distribution of LTL across long-lived and not long-lived subjects, and the distribution of the mean T/S ratio across the various *TERT* and *TERC* genotypes. Level of significance was set at *p* < 0.05. The relationship between T/S ratio and age was evaluated by regression analysis. 

## 3. Results

To evaluate the involvement of the *TERT* and *TERC* polymorphisms in lifespan determination, genotype frequencies of *TERT* and *TERC* SNPs observed in the long-lived subjects were compared against those observed in the subjects who had died before reaching the age of 90 years (not long-lived) (Table 1). In both groups, the genotype frequencies of *TERT* and *TERC* polymorphisms agreed with those expected according to Hardy-Weinberg equilibrium. No difference in the distribution of *TERC* SNPs and *TERT* SNPs rs33954691 and rs2736098 genotypes was observed between the long-lived and the not long-lived (Table 1). By contrast, a significant defect of the *TERT* VNTR MNS16A L/L genotype (*p* = 0.018), and rs2853691 A/G and G/G genotypes (*p* = 0.01), was found in the long-lived compared to the not long-lived. The two *TERT* polymorphisms were found in strict linkage disequilibrium (*p* < 0.0001, D = 80% of Dmax), with a trend of the MNS16A L allele to be associated with the rs2853691 G allele and the MNS16A S allele with the rs2853691 A allele. 

Logistic regression analysis was then applied to correctly evaluate the effect of *TERT* genotypes on longevity. In the analysis, the independent variable was the genotype constituted by the combination of MNS16A L/L and rs2853691 A/G or G/G genotypes. The dependent variable was having lived to an age of over 90 years (≥90 years) or not. The results showed that, after adjusting for sex, carrying MNS16A L/L and rs2853691 A/G or G/G genotypes was associated with a significantly lower probability of living to more than 90 years of age (odds ratio [OR] 0.34, 95% confidence interval [CI] 0.15–0.79, *p* = 0.012), or, in other words, a risk of 2.94 (1/0.34) to die before the age of 90 years. Analysis of the association between *TERT* MNS16A and rs2853691 genotypes and age at death supported previous findings, showing that the L/L genotype and carrying G alleles are associated with a lifespan of less than 90 years (Table 2).

**Figure 1:**
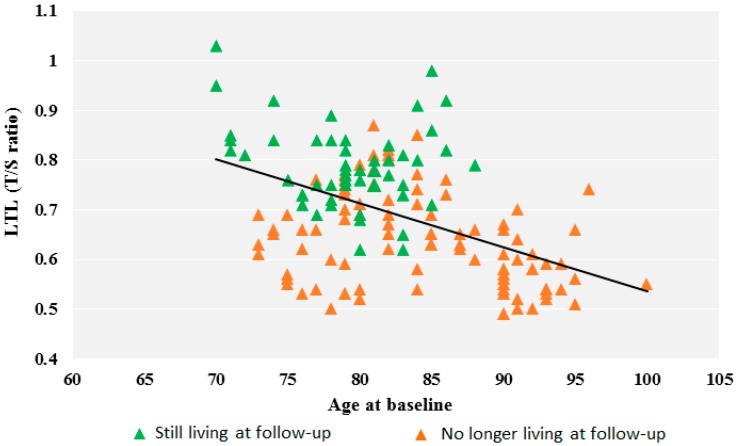
LTL expressed as T/S ratio as a function of age at baseline.

Leukocyte telomere length (LTL), expressed as the T/S ratio, was measured in a subgroup of 153 subjects at baseline. The mean LTL value at baseline was 0.69 ± 0.12 T/S (range, 0.49–1.03, median 0.69), with only a slight difference between males and females (males: *n* = 59, LTL = 0.67 ± 0.11; females: *n* = 94, LTL = 0.70 ± 0.12, *p* = 0.18). Age at baseline was inversely associated with telomere length. Linear regression (*y* = −0.009*x* + 1.4, *p* < 0.0001, *n* = 153) (Figure 1) yielded an estimated telomere loss rate of about 0.010 T/S ratio/year.

Table 3 lists the estimated haplotype frequencies in the long-lived and the controls. In accordance with single polymorphism observations, a significant defect of the MNS16A L—rs2853691 G haplotype was observed in the long-lived compared to the not long-lived to controls (*p* = 0.03), suggesting that the presence of the two *TERT* alleles may prevent attainment of the oldest ages.

The relationship between LTL at baseline and years of life remaining was analyzed using the follow-up data on lifespan. A significant positive relation was observed (regression line *y* = 0.009*x* + 0.6, *p* = 0.01, *n* = 99), where the regression coefficient 0.009 T/S provides an estimate of how much longer the telomeres are at baseline for each additional year of life remaining. We then compared the baseline LTL values of the subjects still living at follow-up with those no longer living in both the total sample, and when the sample was divided into three age classes at baseline (70–79, 80–89, and ≥90 years). The mean LTL values at baseline were significantly higher in those still living than in those who had died during the follow-up years. Within each age group, the LTL of those still living was higher than the mean class value and the LTL of those who had died was lower (Table 4). The difference between those who were still living and those who had died was greater among the 70–79-year-olds than among the 80–89-year-olds (Table 4 and Figure 1). The over 90-year-olds had all died during the follow-up period. 

These findings are illustrated in Figure 1: the vast majority of the deceased in the age range 70–79 years had LTL values below the regression line at baseline, whereas those still living had LTL values above the line. Differently, in the higher age range of 80–90 years, the baseline LTL values of the no–longer living and the still living were fairly mixed below/above the line. We then compared the LTL values in the not long-lived (0.67 ± 0.09, *n* = 56) and the long-lived (0.59 ± 0.08, *n* = 43, *p* = 0.003). The long-lived sample, 84% of which were already 90 years old at baseline, had a lower mean LTL value than the not-long-lived, who belonged to younger age groups. 

Finally, the mean LTL associated with *TERT* VNTR MNS16A and rs2853691 genotypes involved with lifespan determination was examined in not long-lived and long-lived subjects. No difference in mean LTL was observed among *TERT* VNTR MNS16A and rs2853691 genotypes. However, in subjects carrying the combined risk genotypes MNS16A L/L + rs2853691 G/G or A/G (Table 5), LTL was found to be significantly shorter in the Not long-lived than in the Long-lived (*p* = 0.05). No difference in mean LTL was observed among the genotypes of the other *TERC* and *TERT* SNPs (data not reported).

## 4. Discussion

Here, we investigated a possible association between *TERT* and *TERC* polymorphisms and LTL and lifespan by means of a follow-up study. This study design allowed us to extend the investigation to include a sample for which the lifespan was known, and to distinguish between a sample of subjects definitely not long-lived and a sample of long-lived subjects. Furthermore, all the subjects belonged to the same birth cohort and had experienced similar social and environmental influences. 

Examination of the genetic variation of *TERT* and *TERC* genes showed a significant association between two *TERT* polymorphisms (the minisatellite MNS16A and the SNP rs2853691) and lifespan. Carrying the *TERT* VNTR MNS16A L/L genotype and rs2853691 A/G and G/G genotypes turned out to be associated with an increased risk (2.94) of dying before the age of 90 years, i.e., below the mean life expectancy for subjects living in this geographic area, with an average age of about 83 years at study baseline. The observation was confirmed “in vivo” by mortality data and showed that the same risk genotypes were associated with the shortest lifespan. An association between VNTR MNS16A genotypes and longevity has been observed by Concetti et al. [18], whereas rs2853691 SNP has been reported to belong to a haplotype involved in longevity [13,17]. Our data support these findings and highlight that the MNS16A L/L genotype and rs2853691 A/G and G/G prevent the attainment of longevity. Consistent with this result are previous findings that the MNS16A L/L genotype is associated with an increased risk of Alzheimer’s disease [23] and lower survival in patients with glioblastoma or lung cancer [31,32], and that rs2853691 A/G and G/G are associated with esophageal squamous cell carcinoma [33]. The shorter lifespan associated with *TERT* genotypes would therefore, at least in part, be explained by their involvement in the onset of aging-related diseases. We observed a marginally significant association of the combined risk genotypes of the two *TERT* polymorphisms with shorter LTL in the Not long-lived subjects. Although the sample was quite small, this observation is consistent with a previous work [18] that reported a tendency of greater telomere shortening in elderly subjects with homozygous VNTR MNS16A L/L as compared with the other MNS16A genotypes. Considering that the L allele seems to have a negative regulatory role in the expression of telomerase [20], the overall picture suggests that the relationship of *TERT* genotypes with lifespan is mediated by an action of *TERT* on telomere length. We found no significant relationships of the *TERC* SNPs with longevity or telomere length, although the *TERC* SNPs we examined were often found to be associated with telomere length [14,16,17,19], and with longevity (rs3772190) [17]. The conflicting data might depend on diverse factors such as sample size, population examined, and mean age of the population sample, among others. Telomere length being a complex character, numerous genes will contribute to its determination, each providing a small contribution that could be difficult to distinguish. In addition, the interaction of genetic determinants with environmental factors, such as different population lifestyles, could explain the inconsistencies. Furthermore, the discordant results might depend on the technique used for LTL measurement as well. In the majority of the population studies cited above, the average length of telomeres was measured by Real-Time PCR quantitative analysis (qPCR) or by Southern blot analysis of the terminal restriction fragments, and there is evidence that intra- and inter-laboratory technical variation severely limits the comparability of telomere length estimates between laboratories [34].

Here, we also examined the relationships between LTL and lifespan. A significant negative correlation between age and LTL at baseline was observed, with an estimated telomere loss rate of 0.009 T/S ratio/year. This observation is shared by previous studies [35]; the yearly telomere loss was very similar to the reported value (0.010 T/S ratio/year) [35]. The mortality data provided by the follow-up allowed us to evaluate the relationship between LTL at baseline and the number of the remaining years of life. The positive relationship we observed indicates that the shorter the telomeres at baseline, the fewer the remaining years of life. In line with this result, analysis of the mean LTL values at baseline showed that the mean LTL was much shorter in those who had died within nine years of follow-up than in those still alive at follow-up (Table 4). This is partly due to the fact that among the deceased, all were already 90 years old at baseline (about 36%), and therefore with reduced telomeres according to age. The remaining 64% included subjects who had died before the age of 90 and who, as can be seen from the LTL data in Table 4, had LTL values lower than both the average value of their age class and the average value of those still living at follow-up. This is illustrated in Figure 1, where the LTL values for subjects who died during the follow-up are mostly distributed below the regression line. Taking into account the different age classes (Table 4), a comparison of the mean LTL between those still living and those no longer living at follow-up, showed a greater difference in the 70-to-79-year olds compared with the 80-to-90-year-olds, in which the LTL of the no longer living was closer to the LTL of the still living. Again, this pattern is clearly shown in Figure 1: before the age of 80 years, the LTL values of the still living and the no longer living are well-separated by the regression line, but they become quite mixed after the age of 80 years. On the whole the picture provided by our data indicates that telomere length is related to lifespan. Indeed, it is almost a lifespan biomarker. However, this relationship is stronger for the younger age group (70–79), and then weakens after 80 years of age. 

Despite the mixed results [36] of epidemiologic studies investigating the link between LTL and lifespan/mortality, accumulating data tend to confirm that shorter baseline TL is a marker of greater susceptibility to age-related diseases and of higher overall mortality risk [37,38,39]. Varying sample sizes and other characteristics, such as age range or the length of the follow-up period, underlie the conflicting data. In addition, the wide inter-individual variability in telomere length for individuals of the same chronological age due to inherited and environmental factors may mask any relationships between LTL and lifespan [40]. 

The association between TL and lifespan we observed seemed to weaken in the older age classes. Several studies have reported that the magnitude of the association of shorter LTL with higher mortality rates declines with increasing age [4,38,39,40,41,42,43]. A plausible explanation is the so-called “survival bias”. In collecting study samples of older individuals, subjects with shorter baseline TL, being more susceptible to age-related diseases, may be less likely to be included in the study [36,37]. This would lead to a reduced variability of LTL measurements, shifted towards a longer telomere length. Furthermore, leukocyte telomere shortening reflects active cell proliferation triggered by factors such as oxidative stress and chronic systemic inflammation, both of which are aging-related processes. The overexpression of proinflammatory cytokines and mediators observed in older individuals, activating leukocyte proliferation, may lead to alterations in the relationship between LTL and age, and ultimately lifespan [38,44,45]. In this context, it could be hypothesized that, at an age before 80 years, shorter telomere length may indicate a greater susceptibility to aging-related diseases and therefore be predictive of reduced lifespan, whereas in those older than 80 years of age, chronic systemic inflammation, together with oxidative stress, could act as prevailing determinants of leukocyte proliferation and telomere shortening, thus making the relationship between telomere length and lifespan less linear. LTL therefore seems to be a more reliable lifespan biomarker in a younger age class. 

There are indications that the relationship between LTL and age depends on nongenetic factors such as age, sex, race/ethnicity, lifestyle practices, and dietary patterns [10]. The strength of the present paper is the examination of a fairly well-defined population sample for ethnicity, age range, lifestyle, and dietary patterns. This homogeneity allowed us to define a temporal window, the interval of 70–79 years, in which LTL could be seen as a good lifespan biomarker. These observations provide useful indications for designing investigations that aim to assess the possible use of LTL as a lifespan predictor.

## Figures and Tables

**Table 1:** *TERT* and *TERC* genotype distribution in long-lived and not long-lived. Percentage is given in brackets.

Gene/Genotype	Not Long-Lived	Long-Lived
*TERC* rs12696304		
G/G	11 (12.4)	5 (7.0)
G/C	35 (39.3)	31 (43.7)
C/C	43 (48.3)	35 (49.3)
TOTAL	89	71
*p*	0.52	
*TERC* rs3772190		
C/C	56 (67.5)	40 (58.8)
C/T	22 (26.5)	25 (36.8)
T/T	5 (6.0)	3 (4.4)
TOTAL	83	68
*p*	0.40	
*TERC* rs16847897		
C/C	7 (8.6)	8 (11.9)
C/G	36 (44.4)	34 (50.7)
G/G	38 (46.9)	25 (37.3)
TOTAL	81	67
*p*	0.47	
*TERT VNTR*		
*MNS16A*		
L/L L/S ^1^	32 (36.0) 42 (47.2)	13 (18.8) 35 (50.7)
S/S ^1^ TOT	15 (16.9) 89	21 (30.4) 69
*p*	0.018	
*TERT* rs2853691		
A/A	41 (45.1)	48 (67.6)
A/G^1^	42 (46.2)	22 (31.0)
G/G^1^	8 (8.8)	1 (1.4)
TOT	91	71
*p*	0. 004	
*TERT* rs33954691		
C/C	77 (85.6)	62 (84.9)
C/T^1^	11 (12.2)	9 (12.3)
T/T^1^	2 (2.2)	2 (2.7)
TOTAL	90	73
*p*	0.91	
*TERT* rs2736098		
C/C	56 (64.4)	52 (72.2)
C/T^1^	30 (34.5)	17 (23.6)
T/T^1^	1 (1.1)	3 (4.2)
TOTAL	87	72
*p*	0. 29	

^1^ These genotypes were pooled for the analysis.

**Table 2:** Relationship between *TERT* genotypes and age at death (mean ± SD). In brackets is the number of subjects.

SNP/Genotypes	Age at Death
VNTR MNS16A	
L/L	87.6 ± 6.0 (45)
L/S	88.4 ± 5.4 (77)
S/S	90.8 ± 6.2 (36)
*p*	0.04
rs2853691	
A/A	89.9 ± 5.7 (89)
A/G	87.6 ± 5.7 (64)
G/G	85.8 ± 3.7 (9)
*p*	0.01

**Table 3:** *TERT* VNTR MNS16A and *TERT* rs2853691 haplotype distribution in long-lived and not long-lived.

VNTR MNS16A/rs2853691 Haplotype	Not Long-Lived	Long-Lived
L-A	0.294	0.278
L-G	0.301	0.164
S-A ^1^	0.397	0.548
S-G ^1^	0.008	0.010
*p*	0.03	

^1^ These genotypes were pooled for the analysis.

**Table 4:** Mean LTL (T/S ratio) in the total sample, still living and no longer living at follow-up, by age class (mean ± SD).

	All Ages	Age 70–79 Years	Age 80–89 Years	≥ 90 Years
Total sample	0.69 ± 0.12 (153)	0.72 ± 0.12 (54)	0.73 ± 0.10 (63)	0.57 ± 0.06 (36)
No longer living at follow-up	0.63 ± 0.09 (99)	0.63 ± 0.08 (26)	0.69 ± 0.09 (37)	0.57 ± 0.06 (37)
Still living at follow-up	0.79 ± 0.08 (54)	0.80 ± 0.08 (28)	0.77 ± 0.09 (26)	/
*p* ^1^	<0.0001	<0.0001	0.0007	

^1^ The *p* value refers to the comparison between No longer living and Still living at follow-up.

**Table 5:** Mean LTL (T/S ratio) associated with the combined genotypes of MNS16A/rs2853691 polymorphisms.

	L/L + G/G or A/G	L/S or S/S + A/A
Not long-lived	0.63 ± 0.08 (16)	0.64 ± 0.11 (40)
Long-lived	0.72 ± 0.10 (6)	0.66 ± 0.06 (37)
*p*	0.05	0.57

## Supplementary Materials

The following are available online at http://www.mdpi.com/2073-4425/10/2/82/s1, Figure S1: Genotyping of TERT MNS16A; Figure S2: Genotyping of TERT rs2853691; Figure S3: Genotyping of TERT rs33954691.
