# Supplementary material for: Analysis of the Association Between TERC and TERT Genetic Variation and Leukocyte Telomere Length and Human Lifespan—A Follow-Up Study
Source: Genes (Basel). 2019 Jan 25;10(2):82. doi: 10.3390/genes10020082 (PMC6409517; doi:10.3390/genes10020082)
Supplement: Supplementary file 1 [file genes-10-00082-s001.pdf]

Supplementary Materials and Methods

Genotyping

TERT VNTR MNS16A was genotyped according to the allelic-specific PCR method as previously reported (Wang et al., 2003). The primers were: forward: 5′AGGATTCTGATCTCTGAAGGGTG 3′ and reverse 5′TCTGCCTGAGGAAGGACGTATG 3′ (Sigma-Aldrich, MO USA). The amplification procedure consisted of an initial denaturing step for 5′ at 95°C followed by 35 cycles for 1 min a 95°C,1 min a 60°C and 1 min a 72°C and a final extension step for 10 min a 72°C. The PCR products were visualized on 2.5% agarose gel containing 0.25 mg/ml ethidium bromide (Figure S1), genotypes were classified as previously reported (Wang et al., 2003): short allele (S) corresponds to 243 and 274 bp bands and long allele (L) corresponds to 302 and 333 bp bands. Thus, the MNS16A genotypes were: L/L, L/S and S/S.

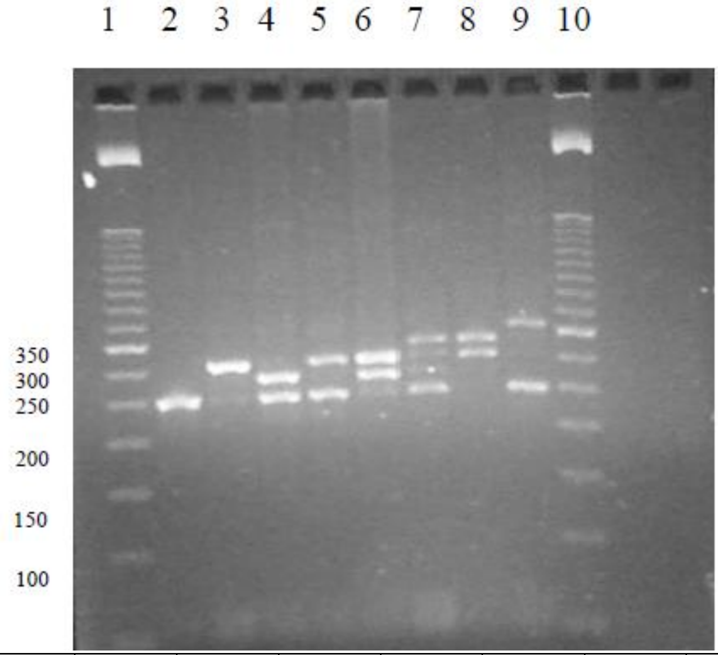

Lanes	2	3	4	5	6	7	8	9
Genotype	243/ 243	302/ 302	243/ 274	243/ 302	302/ 274	243/ 333	302/ 333	243/ 364
Classification	S/S	L/L	S/S	S/L	S/L	S/L	L/L	S/L

Figure S1. Genotyping of TERT MNS16A. Lanes 1, 10: 50 bp ladder. Lanes 2-9: see table.

TERT SNPs (rs2853691 and rs33954691) were investigated by polymerase chain reaction amplification followed by restriction fragment length polymorphism (PCR-RFLP).

The primers for TERT rs2853691 were forward: 5′GGGTAGGGGATAGACAGTGGGA 3′and reverse: 5′CCAGGCTTCCCCATCTTCCCCG 3′ (Sigma-Aldrich, MO USA). The amplification procedure consisted of an initial denaturing step for 5′ at 95°C followed by 35 cycles for 1 min a 95°C,1 min a 60°C, 1 min a 72°C and a final extension step for 10 min a 72°C. The PCR product was digested overnight by the restriction enzyme NciI and then visualized on 2.5% agarose gel (Figure S2).

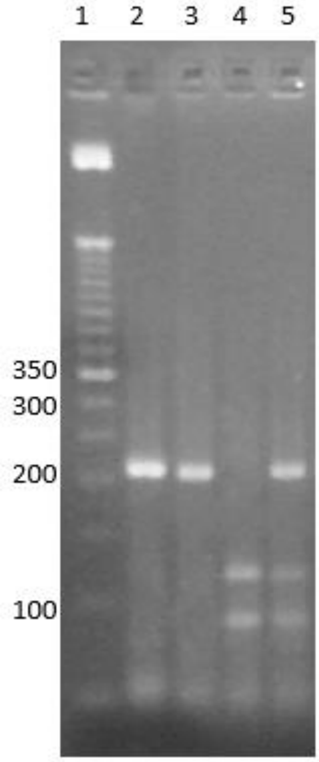

Figure S2. Genotyping of TERT rs2853691. Lanes 1: 50 bp ladder. Lane 2: PCR product, 211 bp. Lane 3: genotype A/A, 211 bp. Lane 4: genotype G/G, 121, 90 bp. Lane 5: genotype A/G, 211, 121, 90 bp.

The primers for TERT rs33954691 were forward: 5′CATTCATGCACGCACACAGGCA3′and reverse: 5′ TCCTAAGCCCAGATT CACTCA 3′ (Sigma-Aldrich, MO USA).The amplification procedure consisted of an initial denaturing step for 5′ at 95°C followed by 35 cycles for 1 min a 95°C,1 min a 56°C,1 min a 72°C and a final extension step for 10 min a 72°C. The PCR product was digested overnight by the restriction enzyme NsiI and then visualized on 2.5% agarose gel (Figure S3).

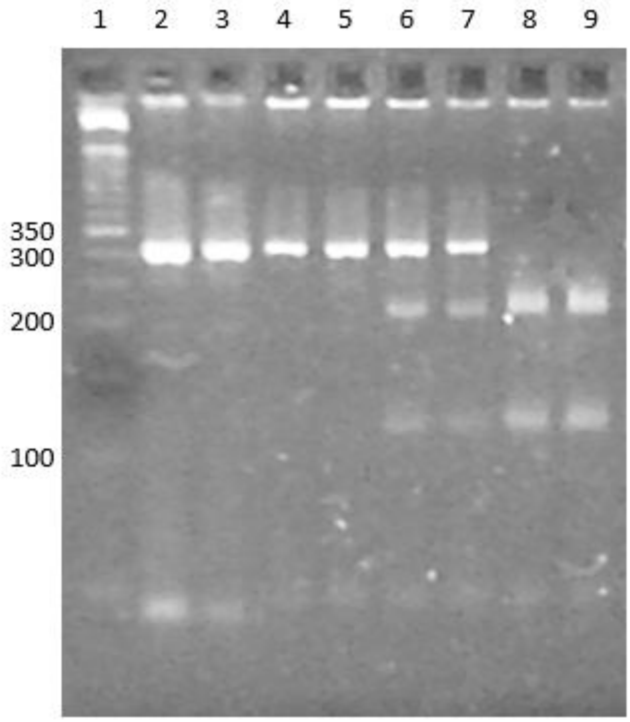

Figure S3. Genotyping of TERT rs33954691. Lane 1: 50 bp ladder. Lanes 2, 3: PCR product, 313 bp. Lanes 4, 5: genotype C/C, 313 bp. Lanes 6, 7: genotype C/T, 313, 205, 107 bp. Lanes 8, 9: genotype T/T, 205, 107 bp.

Genotyping of the TERC SNPs (rs12696304, rs3772190 and rs16847897**)** and TERT SNP rs2736098 was carried out by allelic discrimination using predesigned TaqMan SNP genotyping assays (Applied Biosystems, Carlsbad, CA, USA). DNA was amplified in a total volume of 20 µl containing 10 µl of TaqMan Genotyping Master Mix (Applied Biosystems), 36 µM of each primer, 8 µM of each probe, and approximately 100 ng of template DNA. PCR was performed using a 7300 real-time PCR instrument (Applied Biosystems) under standard conditions.
